# Rethinking the words hotspot reservoir and pristine in the environmental dimensions of antimicrobial resistance

**DOI:** 10.1038/s44259-025-00080-9

**Published:** 2025-02-21

**Authors:** Richard Helliwell, Isabel Ewin, Alexander D. Williams, Diane T. Levine, Andrew C. Singer, Sujatha Raman, Carol Morris, Dov J. Stekel

**Affiliations:** 1 School of Geography, University of Nottingham, University Park Campus, Nottingham, UK; 2 Ruralis, University Centre Dragvoll, Trondheim, Norway; 3 School of Biosciences, University of Nottingham, Sutton Bonington Campus, College Road, Loughborough, Leicestershire UK; 4 Laboratory of Data Discovery for Health Ltd, Hong Kong Science and Technology Park, Tai Po Hong Kong, PR China; 5 School of Public Health, University of Hong Kong, Hong Kong, PR China; 6 School of Criminology, Sociology and Social Policy, University of Leicester, Leicester, UK; 7 Centre for Social Development in Africa, University of Johannesburg, Auckland Park, Johannesburg, South Africa; 8 UK Centre for Ecology and Hydrology, Wallingford, Oxfordshire UK; 9 Centre for Public Awareness of Science, Australian National University, Linnaeus Way, Acton ACT 2601, Canberra, Australia; 10 Department of Mathematics and Applied Mathematics, University of Johannesburg, Auckland Park Kingsway Campus, Rossmore, Johannesburg, South Africa

**Keywords:** Environmental microbiology, Antimicrobial resistance

## Abstract

We assess three words commonly used to represent the environmental dimensions of antimicrobial resistance (AMR) – ‘hotspot’, ‘reservoir’ and ‘pristine’ – through two questions: how are these terms used in published research; and how do these terms shape research being conducted? We advocate for the community to reflect on and improve its use of language, and suggest four potentially more productive and precise terms for AMR hazard: prevalence; transmission; evolution and connectivity.

## Introduction

Antimicrobial resistance (AMR) poses a serious global threat to human health^1^, with bacterial AMR being the central topic of scientific and regulatory interest^2^. The One Health paradigm has brought attention to AMR as an interlinked human, animal and environmental issue^3^. In this article we focus specifically on research related to the environmental dimensions of AMR. There are three major threats associated with AMR in the environment. First, from antimicrobial pollution, including both antibiotic^4^ and non-antibiotic chemicals^5^, especially in water environments^6,7^, creating selection^8^ and co-selection conditions^9^. Second, from the presence and transmission of mobile and mobilizable resistance gene(s)^10–12^ through horizontal gene transfer (HGT), ultimately into clinically important pathogens^13–15^. This includes the risk of HGT transferring previously undocumented antimicrobial resistance genes (ARGs)^16^, or giving rise to new combinations of ARGs, conferring and spreading multi-drug or pan-drug resistance^17–19^. Third, from the direct transfer of AMR pathogens to humans via contaminated water^20^, and food^21^ or inadvertent colonization of commensals containing ARGs through environmental exposure^22^.

The environmental dimensions of AMR poses a distinct set of challenges in comparison with the human and livestock contexts. With the latter, research and policy have drawn attention to failures of public and professional knowledge leading to the overuse and prescription of antibiotics, in turn presenting as a solution the promotion of ‘judicious’^23^, ‘prudent’^24^ or ‘rational’^25^ antibiotic prescribing and use behaviors^26,27^. In contrast, research on the environmental dimensions of AMR draws attention to a broader set of open-ended processes, flows and interactions between society, the environment, antimicrobial chemicals and bacterial ecosystems implicated in the rising prevalence of AMR^28–31^.

There are considerable challenges in determining how complex social-environmental systems can be made accessible to rigorous scientific study, as well as practically or politically manageable. It requires decisions about which environmental and AMR entities, interactions, and networks are most important targets for scientific analysis, across scales from molecular to the entire planet^32^. These decisions bring to the fore the importance of scientific language in framing, describing, and representing these processes.

The relationship between language, science, and society is a long-standing topic of research for social scientists and humanities scholars^33^. Within this work, language is a fundamental tool for thinking about and acting in the world. Language used by scientists influences scientific thought processes and designs, hypothesis development, methods and thus, the conclusions we can come to. How we represent the environmental dimensions of AMR thus influences how science is done and understood^34^. This includes what science is funded, where and what is sampled, choices over data processing and analysis, and consequently, the picture that is collectively developed through rigorous scientific research about the environmental dimensions of AMR, the challenges it poses to human and animal health, and the need for potential surveillance, mitigatory and regulatory actions.

A prominent example of the study of use of language in AMR is the use of war or eschatological metaphors such as the ‘war on superbugs’ or the ‘antibiotic apocalypse’ within public media and science communication^35–37^. But other terminology used to represent microbes and antimicrobial resistance, have largely escaped broader scrutiny^38^. In this piece we specifically study the terms ‘hotspot’, ‘reservoir’ and ‘pristine’ identified in previous work as significant representations in the field^39^. These words sit within a broad spectrum of descriptive scientific language that extends from the precise, e.g., ‘NDM-1 metallo-beta-lactamase’, to less-precise terminology, e.g., ‘hotspot’. Even widely used terms such as AMR, antibiotic resistance or drug resistance have different meanings, significance and framings depending on the community and context within which they are used^40,41^. For example, ‘resistance’ can be seen by medical professionals as treatment failure^42^, or by ecologists as a strain having selective advantage^43^. This imprecise language can focus attention towards areas that are thought to be most important, but risks overemphasis of certain sites or obscuring important dimensions of the phenomena and the hazards they present.

This Perspective is specifically aimed at natural scientists who work in the field of AMR in the environment. We wish to provide a launching point for considering the language used to represent the environmental dimensions of AMR and reflect on its broader consequences for both science and governance. This article assesses two key questions in relation to these terms: (1) how does the scientific community (of which we are part) use these terms in our publications; and (2) how do these terms shape research being conducted in the environment?

## Unpacking environmental terminology

We start by providing an initial definition of each of the three terms. This is followed by an analysis and discussion of how they are used in the literature.

## Hotspots

Since the 1950s, epidemiological approaches sought to identify spatial clusters or concentrations of illness or infectious disease^44^. In the 1980s and 90s, AMR research mainly referred to ‘genetic hotspots’^45^. This changed when ‘hotspot’ was more explicitly popularised around the early 2000s with calls to target infectious disease hotspots in global public health policy and research^46^. This concept is a spatial metaphor that represents environments where there is an elevated concentration of pollutants, infectious agents, or possibilities for disease transmission and thus with the highest risk of producing carcinogenic or pathogenic results^46,47^. Regarding the environmental dimensions of AMR, hotspots are areas in which the selection, accumulation, and transmission of AMR genes and bacteria are anticipated to be concentrated. The environmental AMR hotspot does not necessarily refer to heightened possibilities of infection per se, but also includes sites where there is an abundance of both microbial life and antimicrobial pollutants.

## Reservoirs

In medicine, the notion of the disease ‘reservoir’ emerged around the 1900s and was linked to colonial authorities’ efforts to control disease, human populations, and relations between humans and animals^48^. It was more widely popularised in the 1930s due to the development of ecological approaches to understanding disease outbreaks^44^. Its use in relation to AMR dates back to at least the late 1950s^49^. The reservoir conceptualizes a ‘vessel’, or ‘source’, whether this is a population of organisms or an environment, which harbors pathogens and/or transmits them to an at-risk population. The pathogenic potential of the reservoir is always present but nascent, requiring a moment of transmission to mobilize that potential. In the context of AMR, the ‘reservoir’ partially differs, because it is often conceptualized in relation to genes, rather than pathogenic bacteria: non-pathogenic environmental bacteria are a source of diverse resistance genes that can be mobilized into bacterial pathogens^39^. This genetic pool is framed as a latent environmental threat^50^ or ‘resistome’.

## Pristine environments

The idea of ‘pristine’ environments originated in late 19^th^ century narratives about the Americas as having been largely pristine, uninhabited natural landscapes prior to European arrival^51^. Although this is a debunked proposition, the notion of pristine environments has since become important to environmental and biodiversity science^52,53^. ‘Pristine’ environments are thus imagined as untouched by human activity, and in the case of AMR, less exposed to antibiotic, bacterial or genetic pollutants that might result in elevated levels of AMR^39^. Pristine environments are positioned as a comparator, enabling assessment of a natural baseline, background or benchmark against which more contaminated areas can be compared^54^.

## Literature Analysis of the use of Hotspot, Reservoir and Pristine

To assess how these terms are used within published research into the environmental dimensions of AMR, we used a mini-scoping review following PRISMA guidelines (Supplementary Note 1; Supplementary Figure 1; Supplementary Table 1). The review allowed a rapid assessment of appropriate studies suitable for the aims of the research, is transparent and reproducible, but is not comprehensive of the full set of terminology that could have been considered, nor does it consider (relevant) articles that use none of these terms. The mini-scoping review identified 60 articles containing at least one of the key search terms for detailed assessment (Supplementary Table 2).

**Fig. 1:**
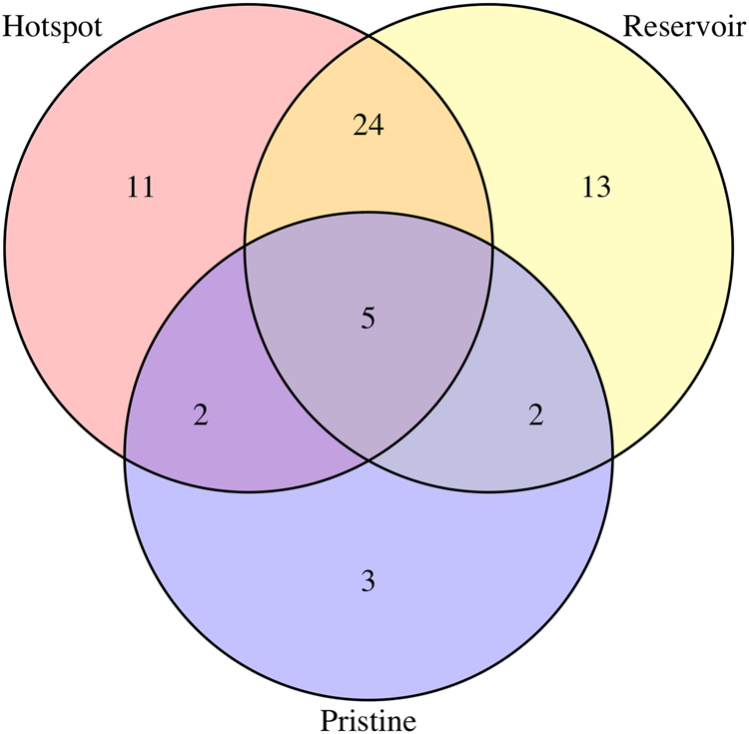
Venn Diagram showing the occurrences of the terms (hotspot, reservoir, and pristine) in 60 studies identified in the mini-review. Hotspot and Reservoir are the more commonly used terms, with many papers making use of both terms.

Hotspot and reservoir are the most popular terms, often being used in the same papers, with pristine being used the least (Fig. 1). Out of the 60 papers 42 used hotspot at least once, 44 used the word reservoir at least once, and 12 used pristine at least once.

## Use of Hotspot in the Literature

‘Hotspot’ appeared in 42 of the 60 articles. The most common use of ‘hotspot’ is to describe a place or area^55^, including pharmaceutical manufacturing sites, sewage and wastewater^56–58^, wastewater treatment plants (WWTPs)^59–71^, biofilm^72^, aquaculture^73^, livestock farms^74^, paddy soils^75^, and slaughterhouses^76^. Thus the term hotspot encompasses a variety of spatial scales from aquaculture or farm sites to biofilms. However, the characteristics of the hotspot can vary: to justify research in a particular location e.g., in a ‘recognized’ hotspot such as a WWTP; as an objective of the work e.g., to identify hotspots within aquatic systems; or as a hypothesis to be tested e.g., slaughterhouses are a potential AMR hotspot^77^. The hotspot is, therefore, also a possible conclusion, the results of analysis confirming or refuting the hypothesis or establishing a new site as a potential hotspot.

Complicating the hotspot further is its use to describe processes. ‘Consumption’ was identified as a hotspot for AMR when, for example, products containing antimicrobials were used, or residues in food could encourage AMR when encountering the human microbiota^55^. ‘Disposal’ was another stage when antimicrobials could be introduced to soil, water and air, stimulating selection and transmission^78^. While these processes map onto the spatial hotspots identified above (farm or WWTP), the emphasis is shifted away from the spatial and towards a set of acts or practices.

In contrast, the notion of certain places as hotspots has been challenged by some scientists due to conflicting empirical results. Rather than confirming the provenance of a hotspot, the presentation of empirical findings from hypothesised ‘hotspots’ often complicates such ready characterization with ambiguous results^79,80^ and the presence of confounding factors^81^. Other work has been more explicit, arguing that WWTPs and animal slurries are not necessarily hotspots^82,83^.

Thus the ‘hotspot’ provides quite different ideas about where and what is important to sample and study. These ideas also communicate different responsibilities and priorities, e.g., emphasising those responsible for antimicrobial pollution, or on individuals entering areas of exposure. Including biofilms as hotspots^72^ raises challenging questions about the scale of study necessary to comprehend the hotspot accurately; is the challenge to comprehend AMR in relation to the farm as a whole, or just the biofilms on said farm? The inclusion of acts of antibiotic consumption and disposal is arguably an effort to refine the hotspot to identify times in which the concentration of antibiotics or potential for AMR selection or transmission is higher at a particular site, such as an aquaculture farm. However, this suggests that antibiotic concentration is the key factor in defining a hotspot, neglecting the enduring effects of consumption or disposal, including accumulation which could be just as significant.

## Use of Reservoir in the Literature

‘Reservoir’ appeared in 44 of the 60 articles. Similar to the hotspot, the reservoir can be presented as a justification and an objective of the work e.g., to identify specific reservoirs within aquatic systems that might be of concern or require more research. Specifically, ‘reservoir’ is used to describe places and organisms where antimicrobial resistant and multi-drug resistant bacterial strains could be isolated in research and from which they could spread further into other environments^84^.

The reservoir is often situated as the background, the ubiquitous presence of microbes that could harbor ARGs, upon which hotspots which might mobilize these ARGS, could be identified. This is especially the case with aquatic systems^61,85–88^, commonly referred to as a reservoir because they harbor taxonomically large and genetically varied systems, with the potential for selection and subsequent transmission of ARGs that could contribute to AMR. Therefore, the task is to understand this aquatic reservoir as a whole and identify the ‘hotspots’ within it, e.g., aquaculture or WWTPs^55,56,73^. Following this, the reservoir is, therefore, sometimes a defined position within a broader system that is situated as a ‘distinct’ reservoir of interest: river sediments^89,90^, surface waters^85^, soil, animal manures and slurries^75,77,91^, or activated sludge^87,92,93^.

Wild animals were also categorized as reservoirs in these articles, be these wild birds^94,95^, wild owls^96^ or wild ungulates^97,98^. These organisms contain distinct microbiota and encounter diverse environments, including those polluted directly or indirectly with antimicrobial pollutants or ARGs. But in contrast to the above framing in which the reservoir is a place, animal reservoirs are organisms or living vectors that circulate ARGs and resistant bacteria between people, agricultural animals and the environment^94^.

A very different and widespread use for ‘reservoir’ is to discuss AMR at a microbiological level. Papers identified Phyla as reservoirs^99^, phages as reservoirs^100^, Bacteroides as reservoirs^65^, and specific microbial species as reservoirs^101^. The reservoir, therefore, attempts to communicate highly varied ideas about the nature of the environmental dimensions of AMR. Is the reservoir everywhere, or is it particular organisms, sites or microbes? Each of these raises quite distinct questions about the type of research necessary, the important dynamics of study and, following this, how the environmental dimensions might be practically managed and understood.

## Use of Pristine in the Literature

Of the 12 articles that used the word ‘pristine’, 8 used it to designate a site to take samples from for comparison with places deemed contaminated^59,90,91,95,102–105^, including rivers or preserved or controlled soils, particularly those that have not been amended with manures containing antibiotics. The assumption is that pristine environments can offer a window into undisturbed microbial communities, allowing us to identify the changes in other locations due to human-caused pollution.

Consequently, pristine appeared most often in relation to methods and research design^102^. This is not to say that pristine means a lack of ARGs, though. Indeed, ARGs are expected to be naturally occurring as reflected in some conceptualizations of the reservoir. Following this, some studies used the concept of pristine to justify sampling in isolated places, for example, Antarctica soil and deep-sea sediments for comparison^90^.

The terminology of a pristine environment was used directly in scientific practice and data interpretation, specifically, how to establish proper comparators and identify control or blank samples. In contrast to the hotspot, which was re-established in new places in response to ambiguous results, the notion of a pristine environment faces a more fundamental challenge to its providence, namely, the difficulty of finding a genuinely pristine place in a polluted world.

## The Uses of Hotspot and Reservoir in the Same Paper

‘Hotspot’ and ‘reservoir’ were both used in 24 papers, the largest point of overlap (Fig. 1). In some articles, reservoir and hotspot were used alongside each other, but are used to describe different spaces and concepts. For example one article^85^ described an aquatic environment as a reservoir with a distinct genetic community; but also as an important place for transmission of AMR genes, a characteristic more often attributed to the hotspot in literature^39^. Some articles have also used these terms to refer to different environments, for example calling a WWTP a hotspot and a biofilm a reservoir^69^; or identifying a clinical facility as a hotspot and describing surface waters as a reservoir^74^.

Other studies use hotspot and reservoir comparably. For example, in the same article, ’hotspot’ is first described as WWTPs^90^, but then biofilms are identified using both ‘hotspot’ and ‘reservoir’, which emphasizes how these terms are interlinked. Other papers use these two terms completely interchangeably^57,106^, e.g., to describe animal farms^98^. That paper, as with many others identified in this study, was written by scientists for whom English might be presumed to be an additional language, which raises additional questions about how these terms are used and understood in a global linguistic context.

## Reflections and Recommendations

Our analysis highlights that there is significant diversity within the representation of the hotspot and the reservoir that is then folded into a singular specification. Indeed, their diverse and imprecise use emphasizes that the meaning and understanding of these terms can be specific to the author, leaving the reader the challenge of interpreting what the author is attempting to portray. Such flexibility is arguably part of the appeal of the words ‘hotspot’ and ‘reservoir’, and their utility for both justifying the locations of scientific study and interpreting conclusions. But it also collapses diverse phenomena with different temporal and spatial scales into a common representation that masks the ontological complexity of the problem of AMR in the environment. Furthermore, we note that although we have examined a sub-section of academic literature, these terms circulate more broadly in scientific and popular material, from newspaper articles^107,108^ to conference posters and presentations, as well as calls for funding.

These representations also seek to enclose the problem, so that AMR is seen as a problem that travels, rhetorically at least, between discrete places, out of an environmental hotspot or reservoir into (say) clinical settings or into otherwise pristine locations, whilst alluding to forms of spatial control that restrict flows between them as the evident solution. Our analysis of ‘pristine’ suggests the incorrectness of this framing, because said locations also consistently contain ARGs and ARBs. AMR is a phenomenon that is produced through interactions between numerous interrelated biological and social drivers^109^, and is therefore challenging to segregate via technological or managerial interventions that do not address these systemic drivers.

Furthermore, there is a risk that ingrained ways of thinking about the world limit our research and potential interventions through narrowed thought patterns arising from uncritical use of language; research based on these ideas may undoubtedly be of value, but we may miss other valuable approaches. One example of how these framings might make a practical difference is in surveillance: the hotspot/reservoir/pristine framing could lead to more focused surveillance on areas conceived as problem or comparator sites; alternative framings could lead to unbiased surveillance programmes e.g., based on a spatial grid. These two framings would produce different evaluations of the prevalence of AMR.

As we have shown, AMR is more complex and varied than can be reasonably described by the representations we have examined in this paper. Based on our analysis, we suggest four terms that could be used more precisely to describe the AMR hazard at sampling locations: *prevalence*, *transmission, evolution*, and *connectivity* (Table 1). Our contention is that these terms could be used either instead of the terms hotspot, reservoir and pristine, amongst other metaphors, or, alternatively, form the basis for justifying or clarifying the use of one or more of those terms, whilst also capturing key facets of AMR as a phenomenon and challenge.

**Table 1:** Definitions of four more specific terms that could be used either as alternatives to, or to justify and clarify, the words hotspot, reservoir or pristine

Term	Description
Prevalence	Measure of ARGs, ARB or Abs at a site as well as hazard associated with specific ARGs and ARB (e.g., resistance to human critical antibiotics).
Transmission	Measure of the hazard of ARGs or ARB being transmitted to humans or animals through exposure.
Evolution	Measure of the hazard of selection for resistance or horizontal gene transfer.
Connectivity	Measure of the macro links or flows between different parts of ecological and human systems.

To show how these terms might be used, we have taken a number of examples from our work and others’ that might fit the description of hotspot, reservoir or pristine, and assessed them against the five criteria we have suggested (Table 2).

**Table 2:** Examples of the application of the four suggested terms to a range of locations or systems that might be described by the terms hotspot, reservoir or pristine: the slurry tank might typically be described as a reservoir; the WWTP or water system in a low income country as a hotspot; and the Arctic as pristine

Scenario/ environment	Prevalence Level of prevalence of ARGs, ARBs or Abs	Transmission Expected likelihood of transmission to humans or animals	Evolution Expected rate of ARG selection or transfer	Connectivity Expected flows e.g., moving water, wild animals, or food
Slurry Tank	High	Low (until spread)	Expected to be high but found to be low	Low in tank; medium when spread
Slurry Amended Soil	Medium	Low	Medium	Medium
WWTP	High	Low	High	High
River downstream of WWTP	High	Medium	Medium	High
Water system in wet, low-lying low-income country	High	High	High	High
Arctic ice	Low	Low	Low	Low

Given the information in Table 2, if one wanted to refer to (say) the slurry tank as a ‘reservoir’, the low-income country water system as a ‘hotspot’ or the Arctic as ‘pristine’, this could now be done while attributing precise meanings to those terms. Alternatively, those terms could be avoided altogether, and replaced with precise terms with measurable attributes.

To conclude, the world is not composed of neatly bound spaces; it contains a continuous gradient of prevalence, hazard, transmission, evolution, and connectivity. Our intention is to establish some potential grounds for clarifying the dynamics of a particular place of study, and the basis for making judgements and assessments about where it sits on such a continuum.

## Supplementary information

Supplementary Information

## Data Availability

This article contains no new data. The list of papers retrieved in the scoping review and data extracted from them are available in Supplementary Table 2.
