# Author Correction: Rethinking the words hostspot reservoir and pristine in the environmental dimensions of antimicrobial resistance

**DOI:** 10.1038/s44259-025-00114-2

**Published:** 2025-05-13

**Authors:** Richard Helliwell, Isabel Ewin, Alexander D. Williams, Diane T. Levine, Andrew C. Singer, Sujatha Raman, Carol Morris, Dov J. Stekel

**Affiliations:** 1 School of Geography, University of Nottingham, University Park Campus, Nottingham, UK; 2 Ruralis, University Centre Dragvoll, Trondheim, Norway; 3 School of Biosciences, University of Nottingham, Sutton Bonington Campus, College Road, Loughborough, Leicestershire UK; 4 Laboratory of Data Discovery for Health Ltd, Hong Kong Science and Technology Park, Tai Po Hong Kong, PR China; 5 School of Public Health, University of Hong Kong, Hong Kong, PR China; 6 School of Criminology, Sociology and Social Policy, University of Leicester, Leicester, UK; 7 Centre for Social Development in Africa, University of Johannesburg, Auckland Park, Johannesburg, South Africa; 8 UK Centre for Ecology and Hydrology, Wallingford, Oxfordshire UK; 9 Centre for Public Awareness of Science, Australian National University, Linnaeus Way, Acton ACT 2601, Canberra, Australia; 10 Department of Mathematics and Applied Mathematics, University of Johannesburg, Auckland Park Kingsway Campus, Rossmore, Johannesburg, South Africa

**Keywords:** Environmental microbiology, Antimicrobial resistance

Correction to: *npj Antimicrobials and Resistance* 10.1038/s44259-025-00080-9, published online 21 February 2025

In the title of this article, the word ‘Hostspot’ was incorrectly spelled and should have read ‘Hotspot’. Additionally, in the Abstract, the word ‘presence’ was incorrectly used and should have read ‘prevalence’, as indicated in Tables 1 and 2. The original article has been corrected.

