# Supplementary material for: Rethinking the words hotspot reservoir and pristine in the environmental dimensions of antimicrobial resistance
Source: NPJ Antimicrob Resist. 2025 Feb 21;3:11. doi: 10.1038/s44259-025-00080-9 (PMC11845593; doi:10.1038/s44259-025-00080-9)
Supplement: Supplementary file 1 — Supplementary Information [file 44259_2025_80_MOESM1_ESM.pdf]

Rethinking the words hostspot reservoir and pristine in the environmental dimensions of antimicrobial resistance

Richard Helliwell[1,2], Isabel Ewin[3], Alexander D Williams[4,5], Diane T Levine[6,7], Andrew Singer[8], Sujatha Raman[9], Carol Morris[1], Dov J Stekel[3,10].

Supplementary Note 1

The scoping review undertaken in this study follow the PRISMA review guidelines[1]. For this review, information was sourced from the Web of Science journal database. This database was searched using one criterion: "antimicrobial resistance" AND ("hot spot*" OR hotspot* OR pristine OR "environmental reservoir*"). The filters for the database were set so the search only captured articles from the past 10 years and in the field of Environmental Science, to keep the study relevant. The search was performed on the 7th November 2022, 86 papers were retrieved for screening, of which 60 were retained for the final review (Figure 2). “Environmental reservoir” was used instead of “reservoir” to narrow down the article output from 250 to 86. In the papers that were selected the use of environmental reservoir and reservoir will be considered due to the similarity in meaning, with reservoir often being used in reference to an environmental reservoir. While we recognize that this has led to exclusion of potentially relevant papers, we do not think that it materially impacts on the key findings.

Article Selection and Data Collection Process

One reviewer assessed all the titles and abstracts including how many times the article included each key word (hotspot, pristine, reservoir). This was followed by a full text reading for the relevant articles by the same reviewer. A table was developed to catalogue the relevant information about the article, this covered: location, sampling method, the aim of the study, the

rationale for the work, and where and how the EIs were used within the paper. There were no processes for obtaining or confirming the data from study investigators as this was deemed not necessary as the review is based primarily on literature. There were no automation tools used in the selection process.

1. Moher, D., Liberati, A., Tetzlaff, J., Altman, D. G. & Grp, P. Preferred reporting items for systematic reviews and meta-analyses: The PRISMA statement. Int. J. Surg. **2010**, *8*, 336-341. https://doi.org:10.1016/j.ijsu.2010.02.007

Supplementary Figure 1 PRISMA process for mini-review

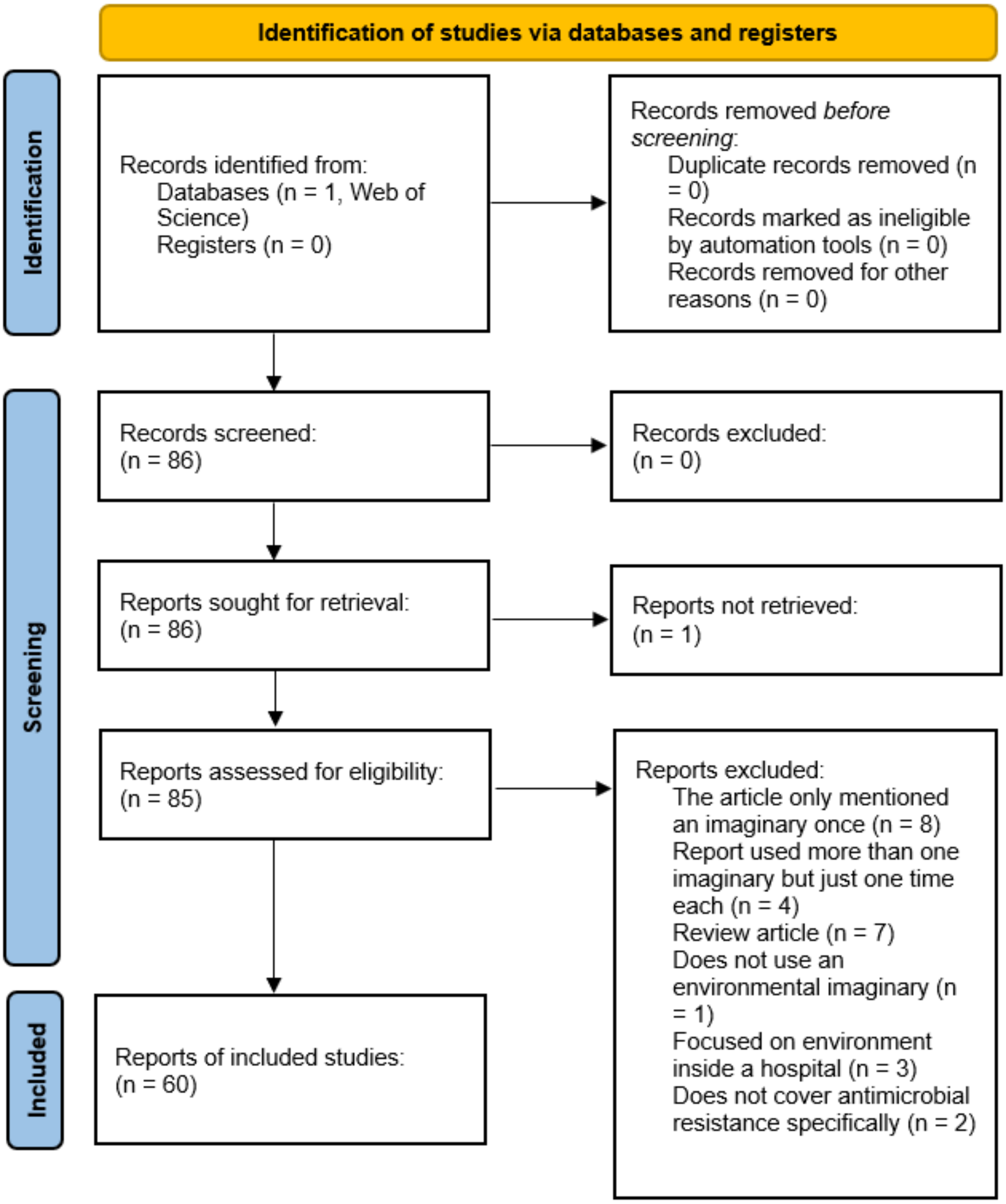

Supplementary Table 1 Inclusion and Exclusion criteria for mini-review

Inclusion	Exclusion
Conducted practical research into antimicrobial resistance in the environment.<br><br>Article looked at a specific gene, microbe, or bacteria.<br><br>Article looked at the genetic community in one location.<br><br>Available in English with full text available.	Article reviewed other studies in the field of antimicrobial resistance.<br><br>Article focused on the hospital environment.<br><br>Article only mentioned one environmental imaginary once in the paper.<br><br>Article mentioned more than one environmental imaginary however they were only mentioned one time each.<br><br>Article did not specifically cover antimicrobial resistance.

Supplementary Table 2 Full list of papers retrieved in the Systematic Review

Primary Author	Year	Title	Global Location	Location in Environment	How many times were each Environmental Imaginary used in each paper			Where in the paper was the Environmental Imaginary used?							
					Hotspot (H)	Pristine (P)	Reservoir (R)	Title	Key Words	Highlights	Abstract	Introduction	Methods	Results & Discussion	Conclusion
Asaduzzaman, M.	2022	Spatiotemporal distribution of antimicrobial resistant organisms in different water environments in urban and rural settings of Bangladesh	Bangladesh	Drinking Water, Wastewater and Surfacewater	2		3				H(1)	R(2)		H(1), R(1)	
Aujoulat, F.	2021	Environmental Antimicrobial Resistance in a Small Urban Mediterranean River: A Focus on Endemic Beta-Lactamases in Clinically Relevant Bacteria	Montpellier, France	Urban Rivers	2		1				H(1), R(1)	H(1)			
Beresin, G. A.	2017	Swine exposure and methicillin-resistant Staphylococcus aureus infection among hospitalized patients with skin and soft tissue infections in Illinois: A ZIP code-level analysis	Illinois, USA	Swine, Agriculture, Hospital			3				R(1)	R(1)		R(1)	
Borsetto, C.	2021	Impact of sulfamethoxazole on a riverine microbiome water research	River Sowe, Stoneleigh, UK	Freshwater Rivers	2		2				H(1), R(1)	H(1)		R(1)	
Brienza, M.	2022	Reclaimed wastewater reuse in irrigation: Role of biofilms in the fate of antibiotics and spread of antimicrobial resistance	France	Reclaimed Wastewater Associated Biofilms	4		4				H(1), R(1)	H(3), R(1)		R(2)	
Brunton, L. A.	2019	Identifying hotspots for antibiotic resistance emergence and selection, and elucidating pathways to human exposure: Application of a systems-thinking approach to aquaculture systems	Hanoi, Vietnam	Aquaculture	30			H(1)		H(1)	H(3)	H(1)	H(3)	H(14),H(6)	H(1)
Calero-Caceres, W.	2019	Antibiotic resistance genes in bacteriophages from diverse marine habitats	Catalan	Marine Habitats	1	2	6		R(1)	R(2)	R(2)	H(1)		P(2)	R(1)

Callahan, M. T.	2019	Salmonella enterica recovery from river waters of the Maryland Eastern Shore reveals high serotype diversity and some multidrug resistance	Delmarva Peninsula, USA	River Water			4				R(1)	R(2)			R(1)
Chen, H. Y.	2019	Characterization of antibiotic resistance genes in the sediments of an urban river revealed by comparative metagenomics analysis	China and Antarctica	Water Sediments		11	3			P(1)	R(1), P(1)	P(1), R(1)	P(3)	P(7), R(1)	
Cheng, X. X.	2021	Metagenomic profiling of antibiotic resistance and virulence removal: Activated sludge vs. algal wastewater treatment system	Las Cruces, New Mexico, USA	Wastewater	1		2		H(1)					R(1)	
Cui, H. L.	2023	Co-occurrence of genes for antibiotic resistance and arsenic biotransformation in paddy soils	China	Paddy Soil	1		3				H(1)			R(3)	
Devarajan, N.	2017	Antibiotic resistant Pseudomonas spp. in the aquatic environment: A prevalence study under tropical and temperate climate conditions	Democratic Republic of the Congo, India, and Switzerland	Water Sediments	1		5				H(1), R(1)	R(1)		R(1), R(2)	
Di Cesare, A.	2022	Contribution of plasmidome, metal resistome and integrases to the persistence of the antibiotic resistome in aquatic environments	Lake Maggiore and River Agogna, Italy and Switzerland	WWTP	3						H(1)	H(2)			
Ewbank, A. C.	2021	Seabirds as anthropization indicators in two different tropical biotopes: A One Health approach to the issue of antimicrobial resistance genes pollution in oceanic islands	Archipelagos off Brazil Coast	Seabirds as Indicators	2	10	2		P(1)	P(1)	P(3)	P(3)		H(2), R(2)	P(2)
Ewbank, A. C.	2022	Extended-spectrum beta-lactamase (ESBL)-producing Escherichia coli survey in wild seabirds at a pristine atoll in the southern Atlantic Ocean, Brazil: First report of the O25b-ST131 clone harboring bla(CTX-M-8)	Archipelagos off Brazil Coast	Seabirds as Indicators		11	3	P(1)		P(1)	P(2)	P(2)		P(5), R(3)	
Fan, H. N.	2021	Characterization of tetracycline-resistant microbiome in soil-plant systems by combination of H(2)18O-based DNA-Stable isotope probing and metagenomics	Luancheng Ecological Station China	Soil-Plant Systems	9		1			H(1)	H(1)			H(2), H(3), R(1)	H(2)
Fuentes-Castillo, D.	2019	Wild owls colonized by international clones of extended-spectrum beta-lactamase (CTX-M)-producing Escherichia coli and Salmonella Infantis in the Southern Cone of America	Southern Chile	Wild Owls			3				R(1)	R(1)			R(1)
Furness, L. E.	2017	Wild small mammals as sentinels for the environmental transmission of antimicrobial resistance	South West England	Wild Small Mammals	1		3		R(1)		H(1)			R(2)	

Goh, S. G.	2022	A new modelling framework for assessing the relative burden of antimicrobial resistance in aquatic environments	Singapore	Natural Aquatic Environments	5					H(1)	H(1)	H(1)			H(2)
Govender, R.	2021	Identification, antibiotic resistance, and virulence profiling of Aeromonas and Pseudomonas species from wastewater and surface water	Durban, South Africa	Wastewater and Surface Water near WWTP	2		1				H(1)			H(1), R(1)	
Guo, J. H.	2017	Metagenomic analysis reveals wastewater treatment plants as hotspots of antibiotic resistance genes and mobile genetic elements	Beijing, China	WWTP: Activated Sludge, Anaerobically Digested Sludge	6			H(1)		H(1)	H(1)			H(3)	
Hassen, B.	2020	Genetic characterization of ESBL-producing Escherichia coli and Klebsiella pneumoniae isolated from wastewater and river water in Tunisia: predominance of CTX-M-15 and high genetic diversity	Tunisia	Wastewater and River Water	2						H(1)	H(1)			
Hayes, A.	2022	Predicting selection for antimicrobial resistance in UK wastewater and aquatic environments: Ciprofloxacin poses a significant risk	UK	Wastewater			2					R(2)			
He, L. Y.	2016	Discharge of swine wastes risks water quality and food safety: Antibiotics and antibiotic resistance genes, from swine sources to the receiving environments	South China	Swine and Wastewater	2	2	1				H(1)	H(1), R(1)	P(1)	P(1)	
Holton, E.	2022	Spatiotemporal urban water profiling for the assessment of environmental and public exposure to antimicrobials (antibiotics, antifungals, and antivirals) in the Eerste River Catchment, South Africa	Eerste River Catchment, South Africa	Urbanwater	5						H(1)	H(2)			H(2)
Hsu, C. Y.	2015	A Potential Association Between Antibiotic Abuse and Existence of Related Resistance Genes in Different Aquatic Environments	Taiwan	River, Sewage off Swine Farms, WWTP, and Reservoirs	3		4				H(1), R(2)	H(1)		R(1)	H(1), R(1)
Kaszab, E.	2021	Groundwater, soil and compost, as possible sources of virulent and antibiotic-resistant Pseudomonas aeruginosa	Hungary	Groundwater, Soil and Compost	3		1				H(1)	H(1), R(1)			H(1)
Kutilova, I.	2021	Extended-spectrum beta-lactamase-producing Escherichia coli and antimicrobial resistance in municipal and hospital wastewaters in Czech Republic: Culture-based and metagenomic approaches	Czech Republic	Hospital Wastewater, Water Downstream, WWTP, River Water	2						H(1)	H(1)			

Labella, A.	2013	High incidence of antibiotic multi-resistant bacteria in coastal areas dedicated to fish farming	Italy	Marine Agriculture			4				R(1)	R(1)		R(1)	R(1)
Leroy-Freitas, D.	2022	Exploring the microbiome, antibiotic resistance genes, mobile genetic element, and potential resistant pathogens in municipal wastewater treatment plants in Brazil	Brazil	WWTP	2						H(1)				H(1)
Li, L. G.	2018	Estimating the Transfer Range of Plasmids Encoding Antimicrobial Resistance in a Wastewater Treatment Plant Microbial Community	Denmark	WWTPs, Activated Sludge			2				R(1)	R(1)			
Lorenzo, P.	2018	Antibiotic resistance in urban and hospital wastewaters and their impact on a receiving freshwater ecosystem	Zenne River, Belgium	Hospital and Urban Wastewater	4						H(1)	H(1)		H(1)	H(1)
Lu, J. Q.	2021	Responses of sediment resistome, virulence factors and potential pathogens to decades of antibiotics pollution in a shrimp aquafarm	Ningbo, China	Aquaculture Shrimp	3	3					H(1)	H(1), P(1)		H(1), P(1)	P(1)
Makowska, N.	2016	Class 1 integrase, sulfonamide and tetracycline resistance genes in wastewater treatment plant and surface water	Łężyca, Poland	WWTP and Surface Waters	2		1				H(1)	H(1), R(1)			
Makowska, N.	2020	Occurrence of integrons and antibiotic resistance genes in cryoconite and ice of Svalbard, Greenland, and the Caucasus glaciers	Greenland, and the Caucasus glaciers	Cryoconite of Ice and Glaciers	1	4	1				P(1), R(1)	P(2), H(1)		P(1)	
Manoharan, R. K.	2021	Shotgun metagenomic analysis reveals the prevalence of antibiotic resistance genes and mobile genetic elements in full scale hospital wastewater treatment plants	Daegu, South Korea	Hospital WWTPs	4		1				H(1)	H(1)		H(2), R(1)	
Muurinen, J.	2022	Antibiotic Resistomes and Microbiomes in the Surface Water along the Code River in Indonesia Reflect Drainage Basin Anthropogenic Activities	Code River, Indonesia	River Surface Water		4					P(1)	P(1)	P(1)	P(1)	
Paulshus, E.	2019	Diversity and antibiotic resistance among Escherichia coli populations in hospital and community wastewater compared to wastewater at the receiving urban treatment plant	Oslo, Norway	Wastewater	4						H(1)	H(2)		H(1)	
Pruden, A.	2012	Correlation Between Upstream Human Activities and Riverine Antibiotic Resistance Genes	South Platte River Basin, USA	River Water, Small Animal Feeding Operations		13					P(2)	P(6)	P(1)	P(2), P(2)	

Rodriguez, E. A.	2021	Metagenomic analysis of urban wastewater resistome and mobilome: A support for antimicrobial resistance surveillance in an endemic country	Antioquia, Colombia.	WWTP	2		2				H(1)	H(1), R(1)		R(1)	
Roman, V. L.	2021	Abundance and environmental host range of the SXT/R391 ICEs in aquatic environmental communities	France	Aquatic Environmtal Communitties	2		11			R(1)	R(2)	H(1), R(4)		R(3)	H(1), R(1)
Rowe, W.	2016	Comparative metagenomics reveals a diverse range of antimicrobial resistance genes in effluents entering a river catchment	Cambridge, UK	River, WWTP Effluent, Diary Farm Effluent			6				R(3)	R(2)			R(1)
Savin, M.	2020	Antibiotic-resistant bacteria and antimicrobial residues in wastewater and process water from German pig slaughterhouses and their receiving municipal wastewater treatment plants	Germany	Wastewate and Swine	3		3			R(1)	H(1)	R(1)			H(2), R(1)
Schutzius, G.	2019	Antibiotic resistance in fecal sludge and soil in Ho Chi Minh City, Vietnam	Ho Chi Minh City, Vietnam	City fecal sludge and soil			6				R(3)	R(2)			R(1)
Sekizuka, T.	2019	Potential KPC-2 carbapenemase reservoir of environmental Aeromonas hydrophila and Aeromonas caviae isolates from the effluent of an urban wastewater treatment plant in Japan	Japan	Urban Wastewater: WWTP			10	R(1)			R(1)	R(2)		R(2)	R(4)
Suzuki, Y.	2013	Susceptibility of Pseudomonas aeruginosa isolates collected from river water in Japan to antipseudomonal agents	Japan	River Water		2					P(1)				P(1)
Torres, R. T.	2021	Emergence of colistin resistance genes (mcr-1) in Escherichia coli among widely distributed wild ungulates	Portugal	Wild Ungulates			9		R(1)		R(1)	R(4)		R(3)	
Torres, R. T.	2022	A high-risk carbapenem-resistant Pseudomonas aeruginosa clone detected in red deer (Cervus elaphus) from Portugal	Portugal	Red Deer, Wild Ungulates			6		R(1)			R(1)		R(3)	R(1)
Torres, R. T.	2022	A walk on the wild side: Wild ungulates as potential reservoirs of multi-drug resistant bacteria and genes, including Escherichia coli harbouring CTX-M beta-lactamases	Portugal	Wild Ungulates	1		7	R(1)			R(1)	H(1)		R(1), R(3)	R(1)
Tsai, H. C.	2018	Distribution and Genotyping of Aquatic Acinetobacter baumannii Strains Isolated from the Puzi River and Its Tributaries Near Areas of Livestock Farming	Taiwan	Rivers Near Areas with Livestock	2		5				H(1)	R(1)		H(1), R(4)	

Voigt, A. M.	2020	Association between antibiotic residues, antibiotic resistant bacteria and antibiotic resistance genes in anthropogenic wastewater - An evaluation of clinical influences	Germany	Wastewater	2		2				H(1)			H(1)	R(2)
Wang, M. Y.	2017	Stepwise impact of urban wastewater treatment on the bacterial community structure, antibiotic contents, and prevalence of antimicrobial resistance	Jinan China	WWTP Processes			2					R(1)		R(1)	
Waseem, H.	2019	POTENTIAL DISSEMINATION OF ANTIMICROBIAL RESISTANCE FROM SMALL SCALE POULTRY SLAUGHTERHOUSES IN PAKISTAN	Pakistan	Small Scale Poultry Slaughter House			6				R(1)	R(4)			R(1)
Xiang, Q.	2018	Spatial and temporal distribution of antibiotic resistomes in a peri-urban area is associated significantly with anthropogenic activities	Ningbo, China	Surface Soil	1	7					P(3)	H(1), P(3)		P(1)	
Xin, H. B.	2022	Animal farms are hot spots for airborne antimicrobial resistance	Beijing, China	Animal Farms	3		3	H(1)			R(1)	R(1)		R(1)	H(2)
Zheng, H.	2020	Pyroligneous acid mitigated dissemination of antibiotic resistance genes in soil	China	Soil	3	3	2				P(1)	H(2), R(2), P(1)		H(1), P(1)	
Zhou, L.	2022	Metagenomic profiles of the resistome in subtropical estuaries: Co-occurrence patterns, indicative genes, and driving factors	South China	Esturies	2		1				H(1)	H(1), R(1)			
Zhou, M.	2020	Evolution and distribution of resistance genes and bacterial community in water and biofilm of a simulated fish-duck integrated pond with stress	South China	Water, Biofilms, Duck Manure	2		1				H(1)	H(1)		R(1)	
Zhou, S.	2019	Deciphering extracellular antibiotic resistance genes (eARGs) in activated sludge by metagenome	China	WWTP	2		5				H(1), R(1)	H(1), R(1)		R(1)	R(2)
Zhou, Z. C.	2022	Short-term inhalation exposure evaluations of airborne antibiotic resistance genes in environments	China	Air Samples: Pig Farms, Hospitals and Surburban Areas.	4					H(1)	H(1)	H(1)		H(1)	
